# Stormorken Syndrome Caused by a Novel STIM1 Mutation: A Case Report

**DOI:** 10.3389/fneur.2021.522513

**Published:** 2021-08-02

**Authors:** Li-Jun Jiang, Xue Zhao, Zhi-Yan Dou, Qing-Xiao Su, Zan-Hua Rong

**Affiliations:** Department of Pediatrics, The Second Hospital of Hebei Medical University, Shijiazhuang, China

**Keywords:** stormorken syndrome, STIM1, mutation, thrombocytopenia, tubular aggregate myopathy

## Abstract

**Objective:** To identify the gene mutation of Stormorken syndrome and review the published Stromal Interaction Molecule 1 (STIM1) mutation phenotype.

**Methods:** We described the clinical and molecular aspects of a Chinese female with Stormorken syndrome by laboratory tests, muscle biopsies, and genetic analysis. We used this information to summarize all the mutation sites reported in the literature. We also reviewed the clinical features of published cases with a gain of function mutations of STIM1.

**Results:** A 12-year-old Chinese female presented with skin purpura in the lower limbs and stroke-like episodes. Muscle biopsy and microscopic examination revealed atrophy in her skeletal muscle. Genetic analysis identified a novel heterozygous missense mutation, a c.1095G>C transition (NM_003156.3), which caused a p.K365N amino acid substitution in the protein and affected a STIM1-orai1-activation region (SOAR).

**Conclusions:** The novel variant c.1095G>C transition (NM_003156.3) was located in the SOAR, which expands the phenotypic spectrum of STIM1 variants in human disorders and may define the molecular basis of Stormorken syndrome.

## Introduction

Stormorken syndrome is a rare autosomal dominant genetic disorder with a complex phenotype comprising muscle fatigue, a bleeding diathesis associated with thrombocytopathy or thrombocytopenia, asplenia, miosis, anemia, migraine, dyslexia, ichthyosis, and short stature (1–4). Tubular aggregate myopathy (TAM) is a disorder with the pathologic feature of tubular aggregates in skeletal muscle. It is characterized by progressive muscle weakness and atrophy (5). York platelet syndrome (YPS) manifests as thrombocytopenia and calcium instability in platelets (6). Some scholars believe that Stormorken syndrome and YPS are the same disease (7). Heterozygous mutations in the Stromal Interaction Molecule 1 (STIM1) have been identified as causatives of Stormorken syndrome. Studies have reported that mutations in the STIM1 can cause both TAM and YPS (8).

STIM1 is a protein containing a single transmembrane domain, mainly across the endoplasmic/sarcoplasmic reticulum, but also across the cell membrane. Its N-terminus is located in the endoplasmic/sarcoplasmic reticulum, and the C-terminus is located in the cytoplasm (9). STIM1 monomer binds Ca^2+^ with low affinity, and Ca^2+^ consumption induces STIM1 protein unfolding and dimerization and multimerization. The C-terminal portion of STIM1 consists of three coiled-coil domains CC1, CC2. and CC3, serine/valine (SP) and lysine-rich (K)-rich regions, inhibition domain (ID), and functional TRIP structure. Since the second CC structure is proved to be the smallest segment with the ability to activate the Orai1 channel, it is also called SOAR (STIM1-ORAI1 Activation Region) (10, 11). In this article, we will report a case of Stormorken syndrome caused by a novel mutation in the STIM1 gene, which has not been reported in previous literature.

## Case Report

### The Main Information of This Case

In this case, the blood tests included the full blood count, muscle enzymes, liver function tests, renal function tests, serum electrolytes, C-reactive protein (CRP), erythrocyte sedimentation rate (ESR), coagulation (including prothrombin time [PT], activated partial thrombin time [APTT], thrombin time [TT], and fibrinogen [FIB]), thyroid function tests (including Thyroxine [T4], triiodothyronine [T3], thyroid-stimulating hormone [TSH], free T3, and free T4), routine urine tests (including red blood cell, white blood cell, protein, ketone bodies, urine PH, and urine specific gravity), antinuclear antibody, antineutrophil cytoplasmic antibody, anticardiolipin antibody, complement 3, and complement 4.

### Neuroimaging

A physician performed the bone marrow puncture. A standard brain Magnetic Resonance Imaging (MRI), Magnetic resonance angiography (MRA), and Magnetic Resonance Venogram (MRV) were performed with 1.5 Tesla equipment (Siemens, Germany). The chest CT and ultrasound echography in the abdomen and heart were performed.

### Muscle Biopsy

We explained to the child and her family the necessary and possible risks of the operation. We obtained signed informed consent from the patient's parents. The surgeon performed a muscle biopsy under local anesthesia, taking the left deltoid muscle.

Fresh muscle tissue was quickly embedded in isopentane-liquid nitrogen after freezing, using a frozen slicer at around −22°C, 5 μm continuous section (transverse muscle fibers).

The main materials included Hematoxylin-eosin (HE), Modified Gomori trichrome (MGT), NADH-tetrazolium reductase (NADH-TR), Succinate dehydrogenase (SDH), Adenosine triphosphatase (ATPase PH: 9.4, PH: 4.6), Periodic acid Schiff (PAS), and Oil red O. The histopathological features of the muscle were observed under the microscope, combined with clinical results.

### Genetic Analysis

The targeted exome sequencing (TES) was performed on DNA from peripheral blood. After the processes of fragmenting the genomic DNA, ligating the paired-End adaptor, amplifying, and purifying, the human whole gene exons and the 50bp bases in their adjacent introns were captured by a TES Kit (xGen Exome Research Panel, IDT, USA). The DNA library was performed post-capture amplification and purifying, and then sequenced by the Illumina HiSeq X Ten (Illumina, USA). Sequence data alignments to the human genome reference (hg19) and variants-calling were performed by NextGene V2.3.4 software (Softgenetics, USA). The mean read depth is >100 ×, and it reached 20 × for 96% of the target sequences. Meanwhile, the annotation information was performed by NextGene V2.3.4 and the laboratory's own scripts. This included the conservation of nucleotide bases and amino acids, predictions of the biological functions, frequency of the normal populations (1,000 Genomes, ExAC, dbSNP database, and local specific databases), and the data from HGMD, Clinvar, and OMIM.

The variants of pathogenicity met the Standards and Guidelines for the Interpretation of Sequence Variants published by ACMG in 2015 with HGVS nomenclature. We used NM_145239.3 as the reference sequence of the PRRT2 gene for analysis. We verified potentially pathogenic mutations using Sanger sequencing, Forward primer: 5′- GACCCATGCCAAGAAACAGT−3′; Reverse primer: 5′- GGATCCATGCAGAGAGGAGA−3′, product length 690 bp.

The PCR reaction system is 25 μl: 2.5 μl containing 10 × Taq buffer, 2.0 μl of dNTPs (2.5 mM), 0.25 μl of Taq polymerase, 50 ng of DNA template, 0.5 μlof upstream and downstream primers (5 μmol/L), and adding deionized water to 25μl. PCR amplification: pre-denaturation at 95°C for 5 min, denaturation at 95°C for 30 s, annealing at 60°C for 30 s, extension at 72°C for 30 s, 35 cycles, and finally, an extension for 5 min. The amplified products were purified and analyzed on an ABI 3130 XL-Genetic Analyzer (Applied Biosystems).

## Results

### Clinical Findings

A 12-year-old Chinese female was referred to our pediatric department because she had lower extremity bleeding points for over 2 weeks and a headache for 1 day. Stroke-like episodes had been observed twice. Signs of these were sudden numbness in the right limb, an inability to move, and being unable to speak. All the symptoms resolved without treatment after about 20 min. The test results were as follows: white blood cell (WBC) 10.25^*^10∧9/L, hemoglobin (HGB) 85 g/L, Platelet (PLT) 19^*^10∧9/L. The physical examination was normal, and the patient was independently ambulant.

### Laboratory Tests

Blood tests revealed anemia (HGB 84 g/L), low platelet counts (12 × 10^9^/L), elevated lactate dehydrogenase serum levels (670 U/L), elevated gamma-hydroxybutyrate dehydrogenase serum levels (612 U/L), low C3 serum levels (0.45 g/L), low C4 serum levels (0.06 g/L), and normal calcium serum levels (2.14 mmol/L). Red blood cell counts were low. An antinuclear antibody test was positive, with positive anti-SSA-60KD and positive anti-SSA-52KD. P-ANCA was positive, but both PR3 antibody and MPO antibody tests were negative. ESR 31 mm/h. The white blood cell count was in the normal range (5.34 × 109/L). Serum IgG (12.9 g/L), IgA (1.86 g/L), and IgM (0.98 g/L) were all in the normal range. A Coombs test was negative. Serum albumin (42 g/L), Alanine aminotransferase (12.2 U/L), aspartate aminotransferase (26 U/L), and serum creatinine (50μmol/L) were normal. Fibrinogen (1.8 g/L), prothrombin time (12.9 s), and activated partial thromboplastin time (41 s) were normal.

Bone marrow: Nucleated cell hyperplasia was markedly active. The megakaryocyte maturation was blocked. NAP: 32 points. Iron staining: extracellular iron was negative, intracellular iron was negative. Abdominal ultrasound showed a normal structure of her liver, gallbladder, pancreas, and spleen.

### Imaging Results

Brain and brainstem MRI revealed no abnormal focal areas of altered signal intensity in the cerebral hemispheres, brainstem, and cerebellum.

**Figure 1:**
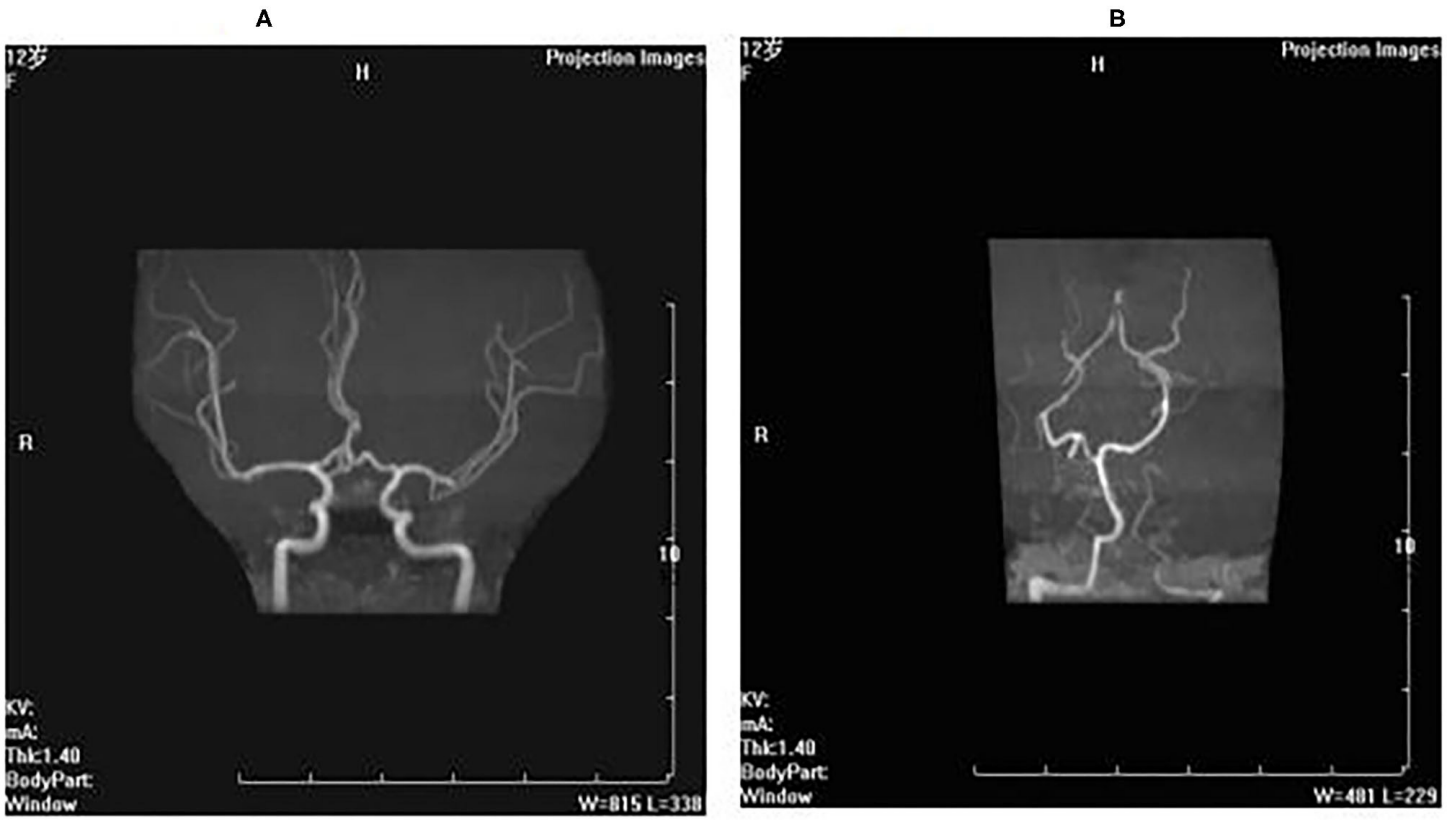
Brain MRA and MRV. **(A)** The brain MRA showed the bilateral intracranial arteries, bilateral anterior cerebral arteries and middle cerebral arteries were stiff, the vascular walls were not smooth with nonuniform signals (vasculitis-like changes). **(B)** The left cerebellar anterior artery extended directly to the left vertebral artery, and the vascular lumen was narrow.

Brain MRA and MRV: The brain MRA showed the bilateral intracranial arteries, bilateral anterior cerebral arteries, and middle cerebral arteries were stiff. The vascular walls were not smooth, with non-uniform signals (vasculitis-like changes) (Figure 1A). The left cerebellar anterior artery extended directly to the left vertebral artery, and the vascular lumen was narrow (Figure 1B).

### Histological Examination

**Figure 2:**
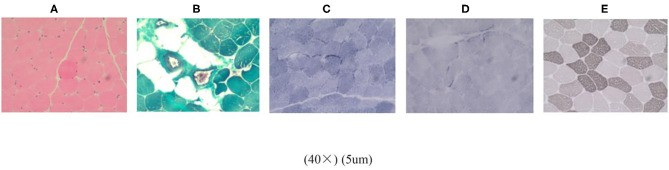
Histological examination of the biopsy of the left deltoid muscle. **(A)** HE staining. **(B)** Modified gomori trichrome staining. **(C)** NADH-TR. **(D)** SDH staining. **(E)** ATPase staining.

A histological examination of the biopsy of the left deltoid muscle revealed atrophic muscle fibers. HE-staining microscope (Figure 2A) showed most of the muscle fibers were normal in size and shape. Some muscle fibers were slightly atrophied, and the atrophic muscle fibers were round or elliptical. In some areas, the muscle fibers had degenerated, but no obvious necrotic muscle fibers were found. Vacuoles were found under the sarcolemma of muscle fibers. PAS staining: particles were found under the membrane of some muscle fibers. Modified Gomori trichrome staining (Figure 2B): the edges of some muscle fibers were slightly red-stained, and no typical broken red fibers were found. NADH-TR (Figure 2C): particle accumulation was found at the edge of type 1 muscle fibers. SDH staining (Figure 2D): particles slightly increased at the edges of some muscle fibers. ATPase staining (Figure 2E): Most type 1 and type 2 muscle fibers were arranged in a mosaic, and some areas were clustered. The number of nerve fibers between the muscle bundles was reduced and not equal in thickness. Oil red O staining: negative. CA staining: negative. No obvious inflammatory cell infiltration or necrosis were observed in the muscle fibers or blood vessels.

In conclusion, the histopathological data obtained from examining the biceps sample revealed some muscle fibers were slightly atrophied. NADH enzyme staining showed muscle fiber sub-membrane granule accumulation. SDH staining showed some muscle fibers' edge particles increased slightly. ATPase staining showed impaired nerve fibers.

### Gene Analysis

**Figure 3:**
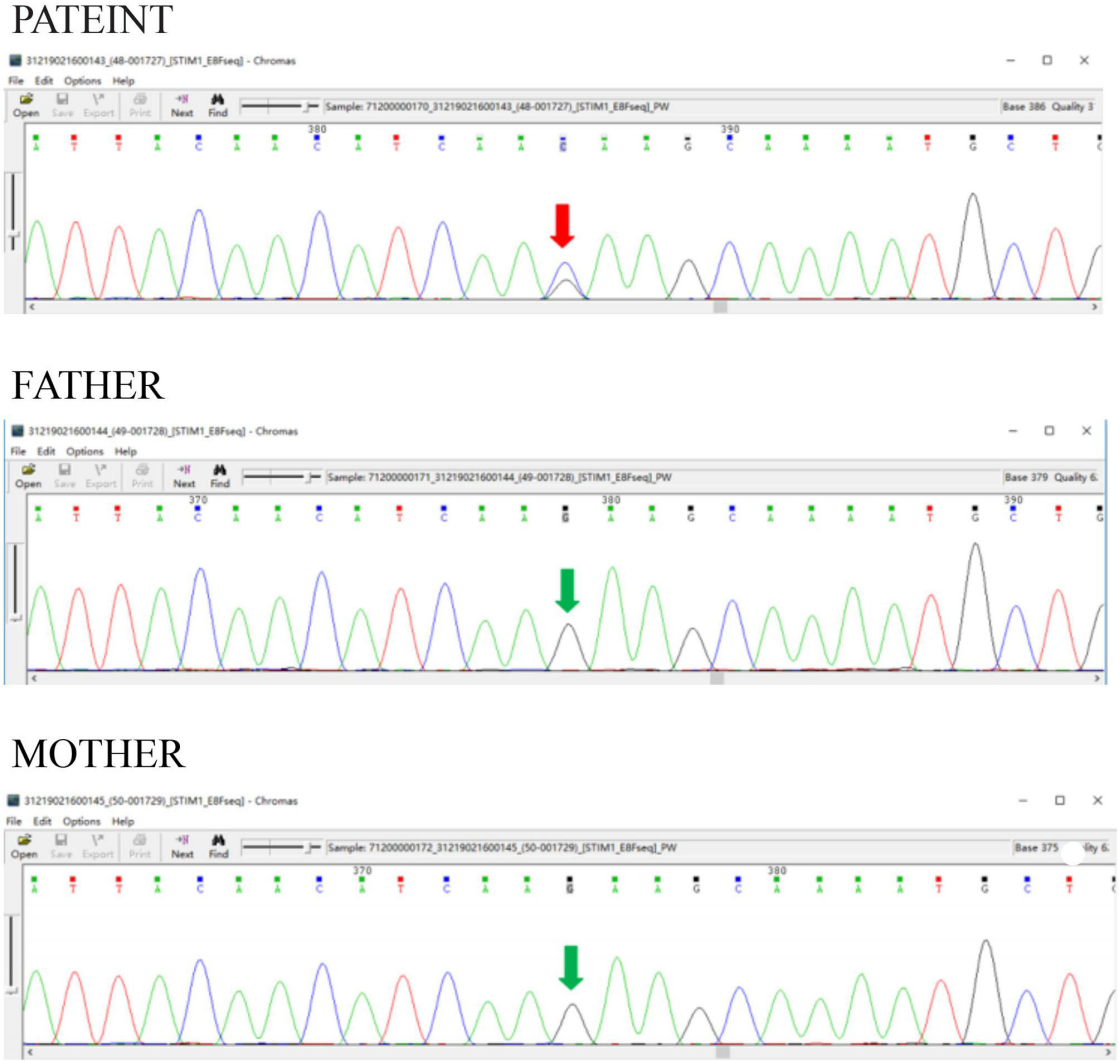
STIM1 gene analysis. STIM1 gene analysis in our patient showed a novel heterozygous missense mutation, a c.1095G>C transition (NM_003156.3), which caused a p.K365N amino acid substitution in the protein and affected a STIM1-orail.

We summarized all the mutation sites reported in previous literature (Table 1) and reviewed the clinical features of published cases with a gain of function mutations of STIM1 (Table 2) (11). According to the previous studies, STIM1 gene analysis in our patient showed a novel heterozygous missense mutation, a c.1095G>C transition (NM_003156.3), which caused a p.K365N amino acid substitution in the protein and affected a STIM1-orai1-activation region (SOAR) (Figure 3). This protein alteration has never been reported in the literature in patients with Stormorken syndrome.

**Table 1:** Summary of the mutation sites reported in previous literature.

**EF1 (*n*)**	**EF2 (*n*)**	**CC (*n*)**	**CTID (*n*)**
c.216C>G (p.H72Q) (3)	c.325C>A (p.H109N) (4)	c.910C>T (p.R304W) (18)	c.1450_1451insGA (p.lle484ArgfsX21) (1)
c.239A>C (p.D80T) (2)	c.326A>G (p.H109R) (6)	c.911G>A (p.R304Q) (3)	
c.242G>A (p.G81N) (3)	c.343A>T (p.I115F) (6)		
c.251A>G (p.D84G) (3)	c.322T>A (p.F108I) (3)		
c.252T>A (p.D84E) (1)			
c.262A>G (p.S88G) (1)			
c.286C>G (p.L96V) (1)			

*N, number of the patients reported in the literature*.

**Table 2:** Clinical features of published cases with gain of function mutations of STIM1.

		**N-terminus mutations**	**C-terminus mutations**
Gender	M	17/33 (52%)	13/22 (59%)
	F	16/33 (48%)	9/22 (41%)
Age of onset	Childhood/adolescence	27/33 (82%)	9/22 (41%)
	Adult	2/33 (6%)	6/22 (27%)
	Not reported	4/33 (12%)	7/22 (32%)
Muscular weakness	+	27/33 (82%)	16/22 (73%)
	-	5/33 (15%)	4/22 (18%)
	Unclear	1/33 (3%)	2/22 (9%)
Miosis	+	7/33 (21%)	16/22 (73%)
	-	4/33 (12%)	6/22 (27%)
	Unclear	22/33 (67%)	0/22 (0%)
CK level	Elevated	31/33 (94%)	22/22 (100%)
	normal	1/33 (3%)	0/22 (0%)
	Unclear	1/33 (3%)	0/22 (0%)
Thrombocytopenia/platelets dysfunction	+	9/33 (27%)	14/22 (64%)
	-	2/33 (6%)	7/22 (32%)
	Unclear	22/33 (67%)	1/22 (4%)
Hypocalcaemia	+	3/33 (9%)	10/22 (45%)
	-	0/33 (0%)	5/22 (23%)
	Unclear	30/33 (91%)	7/22 (32%)
Asplenia	+	5/33 (15%)	11/22 (50%)
	-	2/33 (6%)	7/22 (32%)
	Unclear	26/33 (79%)	4/22 (18%)

## Discussion

We report detailed clinical and genetic findings from a patient with a new STIM1 mutation in the CC domain. Mutations in STIM1 have been thought to result in TAM, Stormorken syndrome, or YPS, depending on the domain affected by the mutations. Our results show that the p.K365N amino acid change caused by c.1095G>C transition of STIM1 affects the function of the SOAR domain, resulting in a gain of function of STIM1. Furthermore, K365N could most probably impair Orai1-activation by STIM1 which cause SOCE.

Calcium (Ca2+) is the second messenger in various cells of the human body. The small disorder of Ca2+ homeostasis can seriously damage the normal physiological functions of various tissues and organs. One of the mechanisms controlling Ca2+ homeostasis is the storage operation of Ca2+ entry (SOCE), which depends on the reticulated Ca2+ sensor STIM1 and the plasma membrane Ca2+ channel ORAI1. SOCE is not restricted to skeletal muscle but also regulates Ca2+ homeostasis in various tissues, explaining the broad phenotypical presentation of Stormorken syndrome associated with STIM1 mutations.

The cytosolic R304W mutation causes constitutive STIM1 clustering and major extracellular Ca2+ influx. The R304W substitution induces a helical elongation within CC1, thereby impeding the inhibitory CC1–CC3 clamp and promoting the exposure of the SOAR domain, resulting in ORAI1 channel activation. There was a less damaging effect of the R304Q mutation on the tight inactive STIM1 conformation, along with the milder clinical phenotype of R304Q compared to patients with R304W mutation. The clinical continuum ranging from a predominant muscle phenotype in patients with EF-hand mutations to the multi-systemic aberrations observed in most patients with the cytosolic R304W mutation might be explained by a different mutational impact on Ca2+-dependent inactivation (CDI). The R304W mutant suppresses fast CDI, that is, the ORAI1 channel inactivation through Ca2+ binding close to the pore.

The clinical manifestations of patients with Stormorken syndrome include muscle weakness, miosis, thrombocytopenia, asplenia, dyslexia, ichthyosis, and short stature. Some scholars believe that Stormorken syndrome and YPS are different names for the same clinical condition (7). Stormorken and TAM are considered to be different stages of the same clinical condition. The pathogenesis of this disease is mainly due to the function-acquired mutation in genes inducing excessive Ca^2+^ into the cell. The main mechanism for controlling Ca^2+^ homeostasis is the store-operated Ca^2+^ entry (SOCE), which depends on the endoplasmic reticulum Ca^2+^ sensor STIM1 and the plasma membrane Ca^2+^ channel ORAI1. Therefore, function-acquired mutations in the STIM1 gene will cause Stormorken syndrome.

Our patient's main clinical manifestation was thrombocytopenia, which was consistent with the Stormorken syndrome reported in the literature. A common feature of patients with Stormorken syndrome and YPS is thrombocytopenia and platelet dysfunction (12). Platelets from patients with Stormorken syndrome are found to be in an activated procoagulant state without prior stimulation, characterized by phosphatidylserine at plasma membranes. The surface of unstimulated platelets from patients with Stormorken syndrome has increased levels of CD63 and CD62p, which are markers of platelet activation (10).

Our patient was anemic and of short stature, which was consistent with previous literature reports. However, this patient had no manifestations of muscle weakness, contracture, hypocalcemia, or asplenia. Her creatine kinase had been normal. Of the 55 cases of Stormorken syndrome reported in the previous literature (2–6, 11, 13–17), one case had a normal creatine kinase range, with muscle pathology suggesting TAM, and the patient had proximal muscle weakness. The gene mutation is c.322T>A(p.F108I) (6). The remaining 54 patients had different degrees of elevated creatine kinases, except one who did not have a detected creatine kinase level (6).

Our patient's muscle pathology suggested muscle atrophy, but there was no typical TAM pathological change. She underwent a head MRI, MRA, and MRV due to a stroke-like episode. The MRA suggested a vasculitis-like change. The change of the intracranial artery might be related to her stroke-like episodes. Regarding the neurological pathology, it was reported that a patient with Stormorken syndrome had corpus callosum agenesis (13). STIM1-mediated SOCE occurs in the central nervous system, and abnormalities in SOCE are associated with multiple signaling pathways and neurological diseases (18).

This patient's autoantibodies (antinuclear antibody, anti-SSA-60KD, and anti-SSA-52KD) were positive, and complement 3 and complement 4 were below normal. Before the genetic test, she was thought to have systemic lupus erythematosus. In previous literature reports, the clinical manifestations of the loss-of-function mutation of the STIM1 gene included immunodeficiency and autoimmune diseases, although autoimmune diseases were not common (19). A loss-of-function mutation of STIM1 showed an autosomal-negative inheritance. There were no cases of autoantibody-positive cases in the function-acquired mutation of the STIM1 gene, which showed an autosomal dominant inheritance. Lymphoproliferation was a common feature of loss-of-function STIM1 mutation in patients. Some patients developed Coombs-positive autoimmune hemolytic anemia (AIHA) or thrombocytopenia in their early life (19). Autoantibodies against platelets resulted in thrombocytopenia. The reason for the STIM1 gene mutations causing positive autoantibody and declined complement needs further study. Is there a STIM1 gene mutation in some patients with SLE? The relationship between STIM1 gene mutation and SLE needs further study. Therefore, patients with low platelets in the clinic should be considered for genetic testing.

This patient was initially treated with hormones and sirolimus because of a suspected diagnosis of SLE. The platelets returned to normal after 1 month of treatment, and hemoglobin returned to normal after 2 months. But at present, there is no specific drug for the disease. Some researchers believe that resveratrol, as an inhibitor of SOCE, may be used to treat the disease (2, 20). With future research on the molecular mechanism of Stormorken syndrome, new effective drugs may be used in clinical cases.

## Conclusions

In summary, the clinical manifestations of STIM1 gene variants are diverse, but the main manifestations are platelet abnormalities and muscle weakness. When encountering such patients in clinical situations, genetic testing can be performed to confirm the diagnosis further.

## Ethics Statement

Written informed consent was obtained for the publication of this case report.

## Author Contributions

L-JJ have made substantial contributions to conception and design. XZ, Q-XS, and Z-YD acquisition of data, analysis, and interpretation of data. L-JJ have been involved in drafting the manuscript and revising it critically for important intellectual content. Z-HR have given final approval of the version to be published. All authors contributed to the article and approved the submitted version.

## Conflict of Interest

The authors declare that the research was conducted in the absence of any commercial or financial relationships that could be construed as a potential conflict of interest.

## Publisher's Note

All claims expressed in this article are solely those of the authors and do not necessarily represent those of their affiliated organizations, or those of the publisher, the editors and the reviewers. Any product that may be evaluated in this article, or claim that may be made by its manufacturer, is not guaranteed or endorsed by the publisher.

## References

[1] Fahrner M Stadlbauer M Muik M Rathner P Stathopulos P Ikura M. A dual mechanism promotes switching of the Stormorken STIM1 R304W mutant into the activated state. Nat Commun. (2018) 9:825. 10.1038/s41467-018-03062-w 29483506 PMC5827659

[2] Borsani O Piga D Costa S Govoni A Magri F Artoni A. Stormorken Syndrome caused by a p.R304W STIM1 mutation: the first Italian patient and a review of the literature. Front Neurol. (2018) 9:859. 10.3389/fneur.2018.00859 30374325 PMC6196270

[3] Harris E Burki U Marini-Bettolo C Neri M Scotton C Hudson J. Complex phenotypes associated with STIM1 mutations in both coiled coil and EF-hand domains. Neuromuscul Disord. (2017) 27:861–72. 10.1016/j.nmd.2017.05.002 28624464

[4] Noury JB Böhm J Peche GA Guyant-Marechal L Bedat-Millet AL Chiche L. Tubular aggregate myopathy with features of stormorken disease due to a new STIM1 mutation. Neuromuscul Disord. (2017) 27:78–82. 10.1016/j.nmd.2016.10.006 27876257

[5] Roman J Palmer MI Palmer CA Johnson NE Butterfield RJ. Myopathy in the york platelet syndrome: an underrecognized complication. Case Rep Pathol. (2018) 2018:5130143. 10.1155/2018/5130143 30159190 PMC6109526

[6] Markello T Chen D Kwan JY Horkayne-Szakaly I Morrison A Simakova O. York platelet syndrome is a CRAC channelopathy due to gain-of-function mutations in STIM1. Mol Genet Metab. (2015) 114:474–82 10.1016/j.ymgme.2014.12.307 25577287 PMC4355183

[7] Singh AR Morin G Rochette J. Stormorken syndrome or York platelet syndrome: a clinician's dilemma. Mol Genet Metab Rep. (2015) 2:80. 10.1016/j.ymgmr.2015.01.003 28649531 PMC5471154

[8] Feske S. Immunodeficiency due to defects in store-operated calcium entry. Ann N Y Acad Sci. (2011) 1238:74–90. 10.1111/j.1749-6632.2011.06240.x 22129055 PMC3774594

[9] Böhm J Laporte J. Gain-of-function mutations in STIM1 and ORAI1 causing tubular aggregate myopathy and Stormorken syndrome. Cell Calcium. (2018) 76:1–9. 10.1016/j.ceca.2018.07.008 30243034

[10] Lacruz RS Feske S. Diseases caused by mutations in ORAI1 and STIM1. Ann N Y Acad Sci. (2015) 1356:45–79. 10.1111/nyas.12938 26469693 PMC4692058

[11] Okuma H Saito F Mitsui J Hara Y Hatanaka Y Ikeda M. Tubular aggregate myopathy caused by a novel mutation in the cytoplasmic domain of STIM1. Neurol Genet. (2016) 2:e50. 10.1212/NXG.0000000000000050 27066587 PMC4817897

[12] Silva-Rojas R Treves S Jacobs H Kessler P Messaddeq N Laporte J. STIM1 over-activation generates a multi-systemic phenotype affecting skeletal muscle, spleen, eye, skin, bones, and the immune system in mice. Hum Mol Genet. (2019) 28:1579–93. 10.1093/hmg/ddy446 30576443

[13] Alonso-Jiménez A Ramón C Dols-Icardo O Roig C Gallardo E Clarimón J. Corpus callosum agenesis, myopathy and pinpoint pupils: consider Stormorken syndrome. Eur J Neurol. (2018) 25:e25–e26. 10.1111/ene.13545 29356264

[14] Li A Kang X Edelman F Waclawik AJ. Stormorken syndrome: a rare cause of myopathy with tubular aggregates and dystrophic features. J Child Neurol. (2019) 34:321–4. 10.1177/0883073819829389 30761937

[15] Nesin V Wiley G Kousi M Ong EC Lehmann T Nicholl DJ. Activating mutations in STIM1 and ORAI1 cause overlapping syndromes of tubular myopathy and congenital miosis. Proc Natl Acad Sci USA. (2014) 111:4197–202. 10.1073/pnas.1312520111 24591628 PMC3964084

[16] Hedberg C Niceta M Fattori F Lindvall B Ciolfi A D'Amico A. Childhood onset tubular aggregate myopathy associated with de novo STIM1 mutations. J Neurol. (2014) 261:870–6. 10.1007/s00415-014-7287-x 24570283

[17] Böhm J Chevessier F Maues De Paula A Koch C Attarian S Feger C. Constitutive activation of the calcium sensor STIM1 causes tubular-aggregate myopathy. Am J Hum Genet. (2013) 92:271–8. 10.1016/j.ajhg.2012.12.007 23332920 PMC3567276

[18] Moccia F Zuccolo E Soda T Tanzi F Guerra G Mapelli L. Stim and Orai proteins in neuronal Ca(^2+^) signaling and excitability. Front Cell Neurosci. (2015) 9:153. 10.3389/fncel.2015.00153 25964739 PMC4408853

[19] Fuchs S Rensing-Ehl A Speckmann C Bengsch B Schmitt-Graeff A Bondzio I. Antiviral and regulatory T cell immunity in a patient with stromal interaction molecule 1 deficiency. J Immunol. (2012) 188:1523–33. 10.4049/jimmunol.1102507 22190180 PMC3262903

[20] Casas-Rua V Alvarez IS Pozo-Guisado E Martin-Romera FJ. Inhibition of STIM1 phosphorylation underlies resveratrol-induced inhibition of store-operated calcium entry. Biochem Pharmacol. (2013) 86:1555–63. 10.1016/j.bcp.2013.09.018 24095720

